# Supplementary material for: Discovery of flat seismic reflections in the mantle beneath the young Juan de Fuca Plate
Source: Nat Commun. 2020 Aug 17;11:4122. doi: 10.1038/s41467-020-17946-3 (PMC7431579; doi:10.1038/s41467-020-17946-3)
Supplement: Supplementary file 1 — Supplementary Information [file 41467_2020_17946_MOESM1_ESM.pdf]

Discovery of flat seismic reflections in the mantle beneath the young Juan de Fuca Plate

Yanfang Qin[1,2], Satish C. Singh[1*], Ingo Grevemeyer[3], Milena Marjanović[1], W. Roger Buck[4]

(1) Institut de Physique de Globe de Paris, 1 rue Jussieu, Paris 75238, France

(2) Now at Japan Agency for Marine-Earth Science and Technology (JAMSTEC), Showa-machi 3173-25, Kanazawa-ku, Yokohama 236-0001, Japan

(3) GEOMAR, Helmholtz Centre for Ocean Research Kiel, Wischhofstr. 1-3

24148 Kiel, Germany

(4) Lamont-Doherty Earth Observatory, Columbia University, 61 Route 9W, Palisades, New York 10964-1000, USA

*Corresponding author: Satish Singh (singh@ipgp.fr)

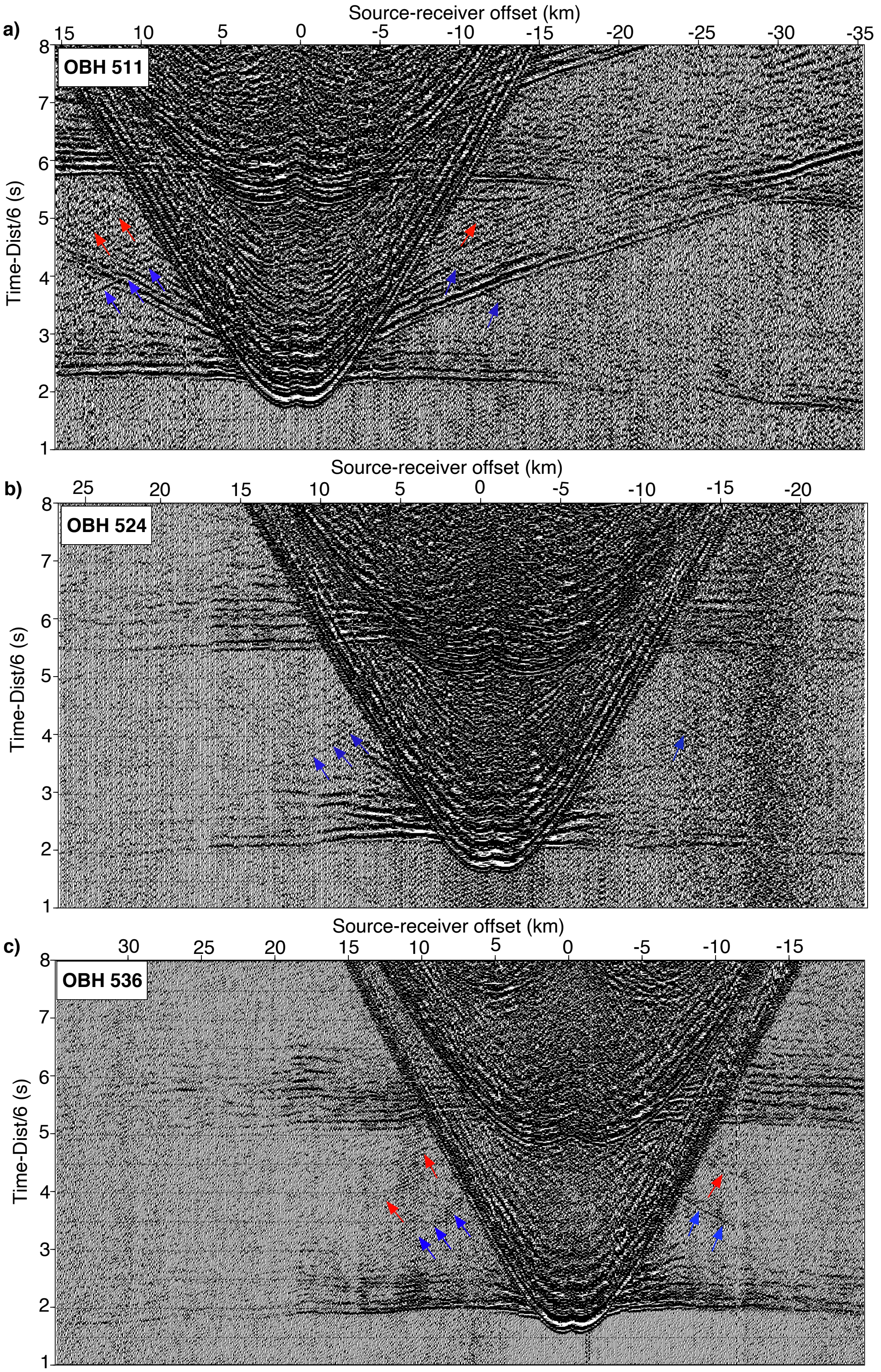

Supplementary Figure 1 | OBH wide-angle seismic data. OBH gathers for profiles RFR96-03 (**a**), 96-05 (**b**), and 96-08 (**c**) are shown for average crustal ages ~1.65, 0.9, 0.51 Ma, respectively. A predictive deconvolution was applied to eliminate some of the reverberations introduced by the bubble pulse signal. The mantle reflection events are marked in blue (shallower) and red (deeper) arrows.

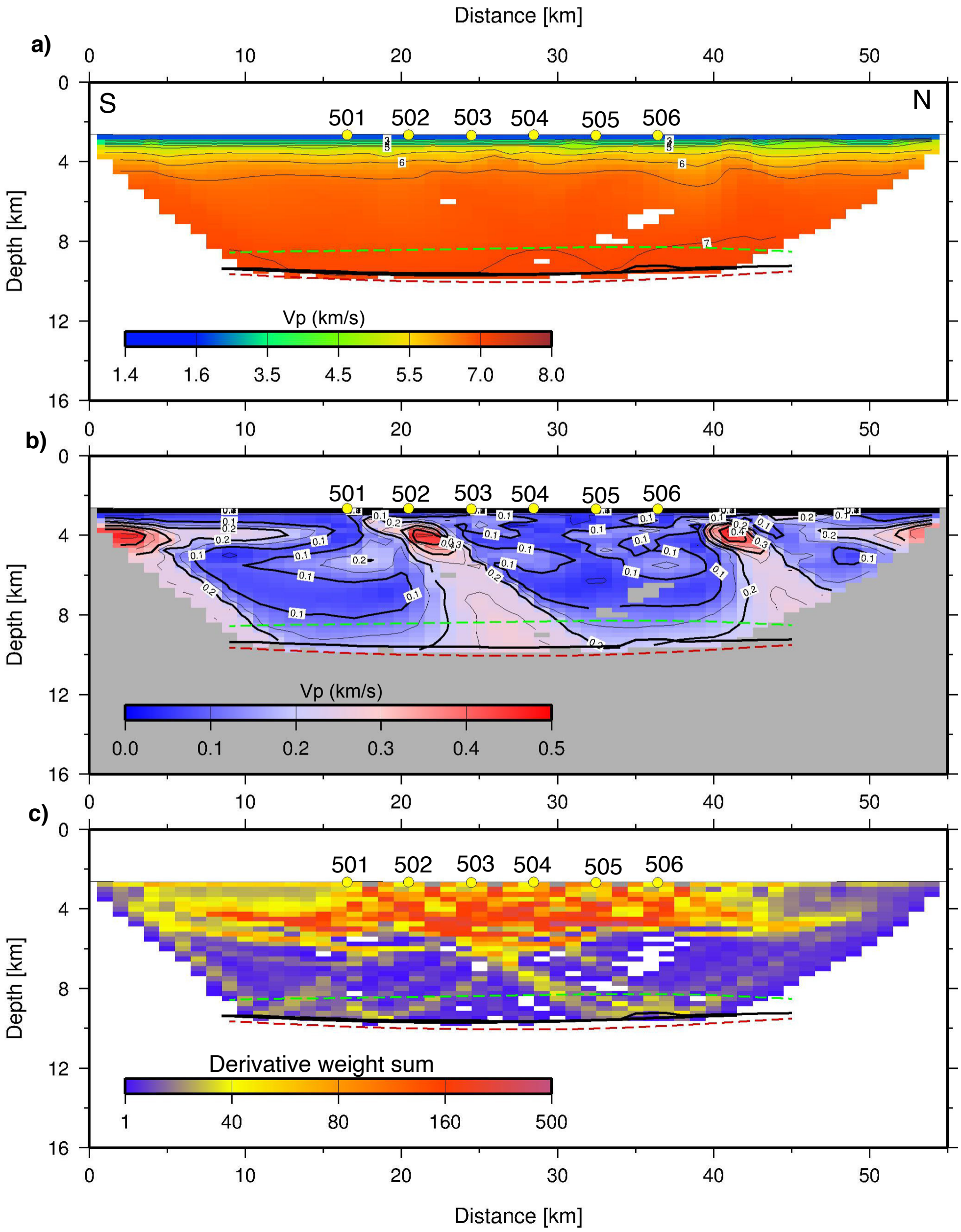

Supplementary Figure 2 | Tomographic velocity model for profile RFR96-01. **(a)** An average inverted velocity model from 100 random 1-D starting models. **(b)** A root mean square uncertainty. **(c)** Derivative weight sum showing the ray coverage. The results are obtained using ray-based travel-time tomography[1] of the crustal (Pg) and Moho reflections (PmP). The location of OBH instruments is shown in yellow circles with their numbers (501-506). Red and green dashed lines indicate upper and lower bounds of starting Moho depth. Note the ray coverage of Pg is down to 3 km depth, and the lower crustal velocity is determined from the PmP arrivals and is poorly constrained.

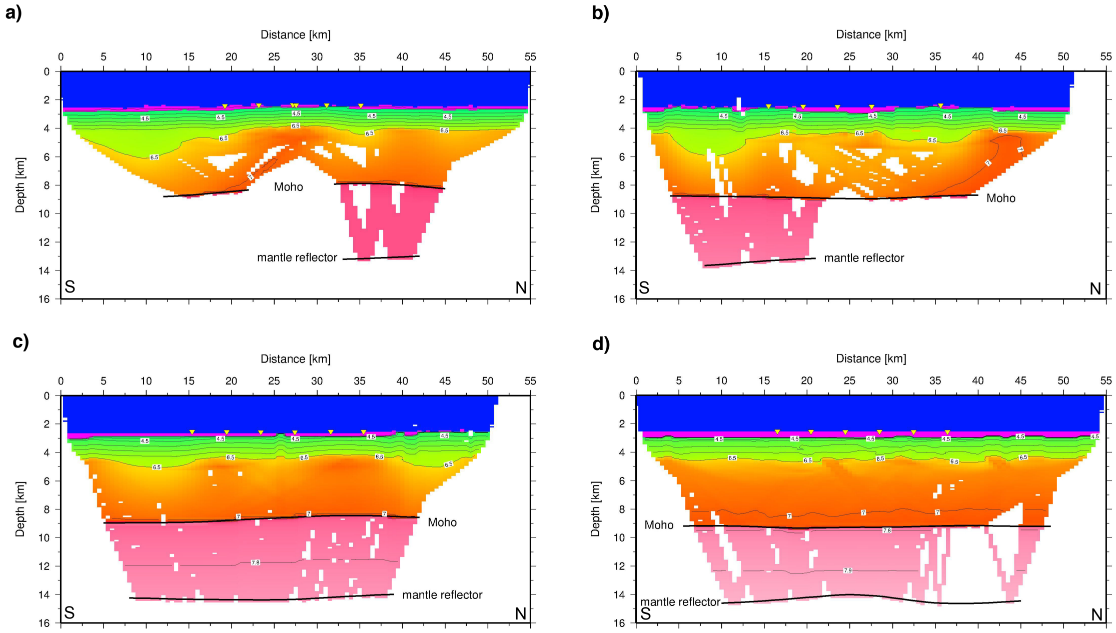

Supplementary Figure 3 | Modelling of mantle reflections. Velocity models for the crust and uppermost mantle obtained using ray-based travel-time tomography. **(a)** Velocity model for profile RFR96-08 sampling the youngest crust. An average upper mantle velocity is 7.60-7.65 km s^{-1}. **(b)** Velocity model along profile RFR96-05 with average velocity in the upper mantle 7.65-7.75 km s^{-1}. **(c)** Velocity model along profile RFR96-03, where upper mantle velocities are 7.7-7.8 km s^{-1}. **(d)** The highest upper mantle velocities ~7.8-7.95 km s^{-1} are mapped along profile RFR96-01 (the profile samples the oldest crustal age in our study area). The average depth of the mantle reflector mapped in the velocity models is at 13.1 km, 13.4 km, 14.3 km, and 14.4 km below the sea surface, respectively. Reverse yellow triangles show the locations of the OBH instruments.

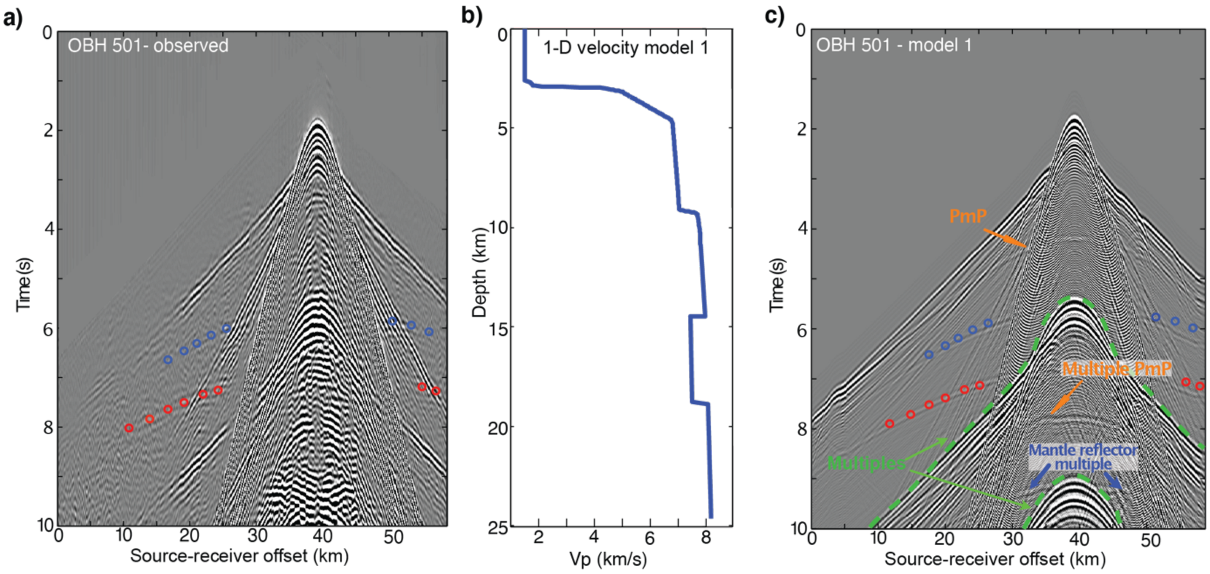

Supplementary Figure 4 | Synthetic seismogram modelling for multiples. (a) Recorded by OBH 501 showing two mantle reflections, marked in blue and red circles. **(b)** 1-D velocity model with a thick low velocity layer embedded in the mantle at 13.5 km depth, and **(c)** the corresponding synthetic seismograms. The seafloor multiples are highlighted in light green. The water bottom multiples of the PmP and mantle reflections are highlighted in orange. Note that the depth in the model starts at the sea surface whereas the OBH is placed on the seafloor in order to mimic the real OBH survey geometry.

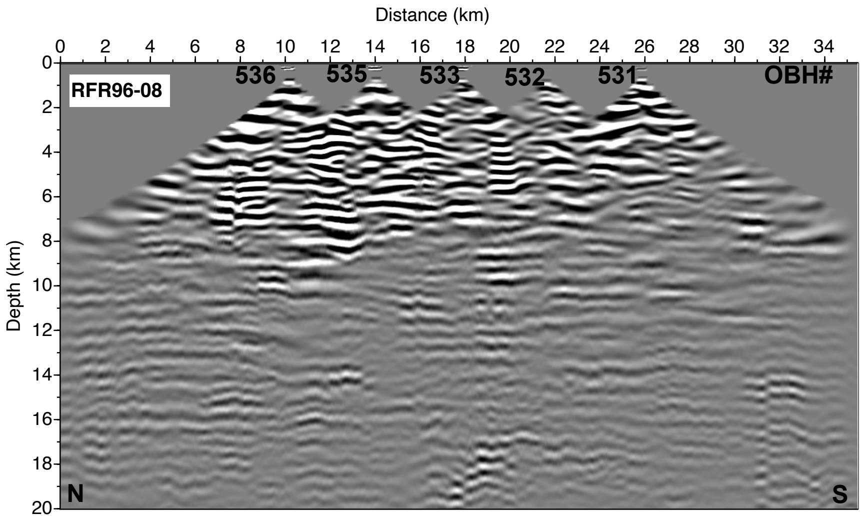

Supplementary Figure 5 | Pre-stack depth migrated seismic image along profile RFR96-08. The OBH instruments are numbered and indicated at the top.

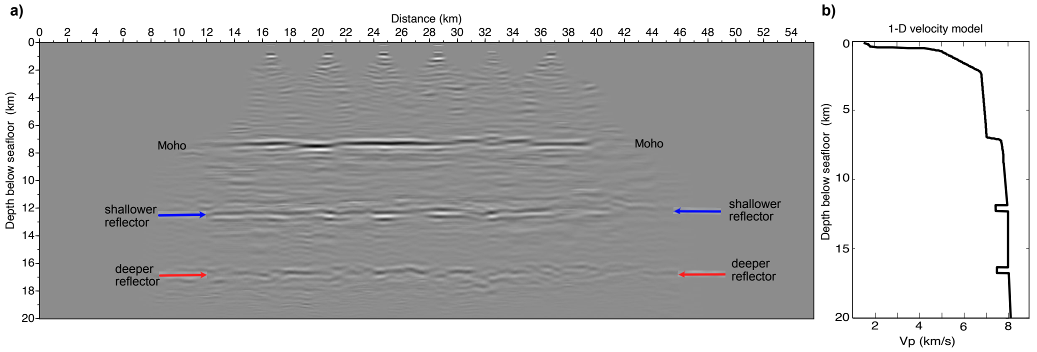

Supplementary Figure 6| Pre-stack migration of synthetic data. (a) Pre-stack depth migrated seismic section using synthetic OBH data. The data were processed following the same steps as for real OBH datasets. In blue and red arrows, we indicate the shallower and deeper reflection events comparable to the ones observed in migrated seismic sections using real OBH data shown in Figure 2. **(b)** The one-dimensional velocity model used to calculate synthetic OBH data.

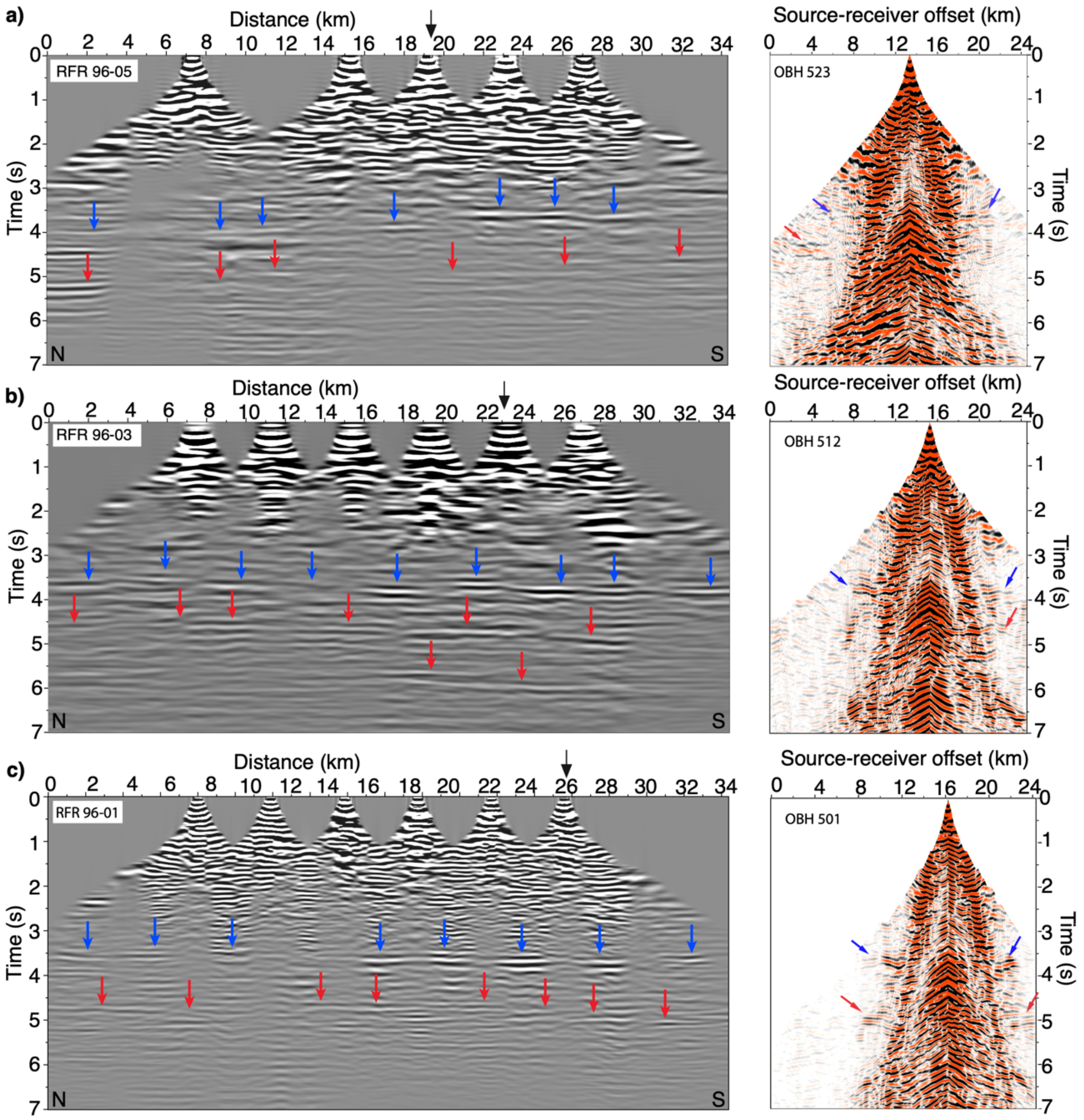

Supplementary Figure 7 | Post-stack time migrated sections of the OBH data. (a) Profile RFR96-05, average crustal age is ~0.9 Ma (note that OBH 525 is missing); **(b)** Profile RFR96-03, sampling crustal age ~1.65 Ma; **(c)** Profile RFR96-01, crustal age is ~2.67 Myr. The blue and red arrows mark shallower and deeper reflection events, respectively. The black arrows mark the locations of OBH gathers shown on the right. The gathers represent normal-moveout (NMO) corrected OBH records after downward continuation the shots to the seafloor level. The colour arrows (blue and red) mark the mantle reflections flattened after NMO, consistent with the bright reflections in the time migrated images.

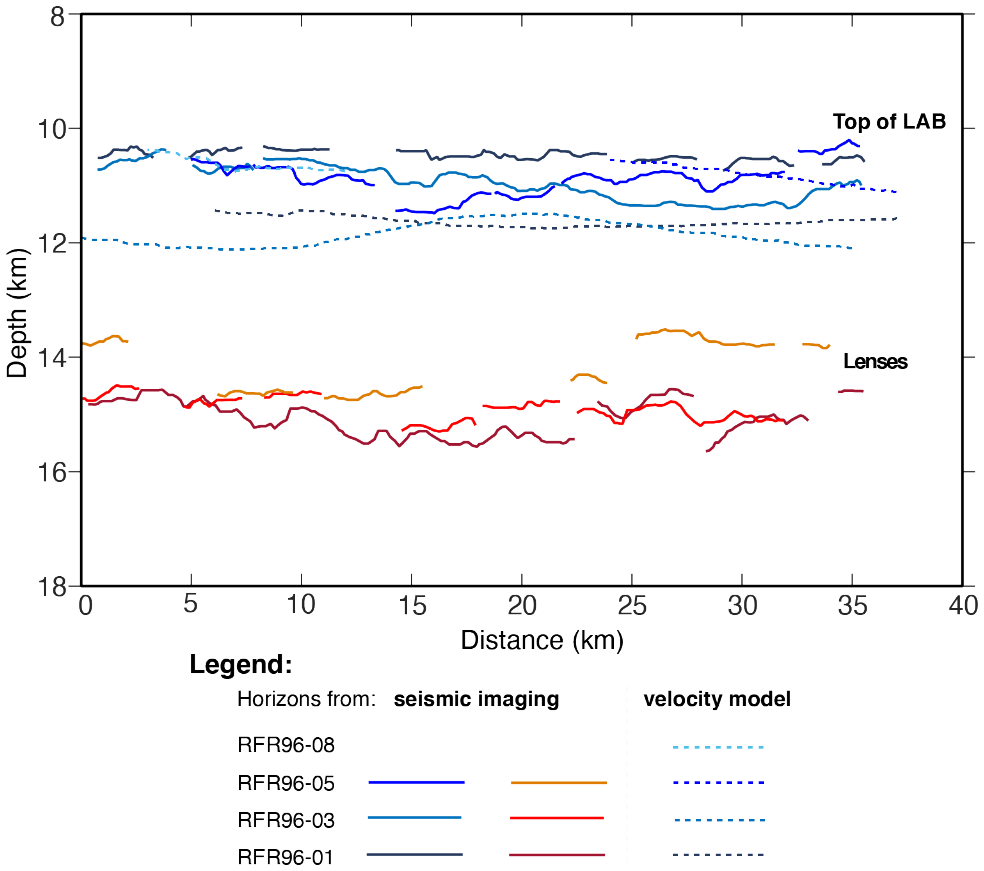

Supplementary Figure 8 | Depth of reflectors. A composite plot showing the depth of the top and bottom reflectors identified along OBH profiles from pre-stack depth migrated images (Fig. 3 and Supplementary Fig. 5) and travel time tomography (dashed; Supplementary Fig. 3). The colour code is shown in Legend.

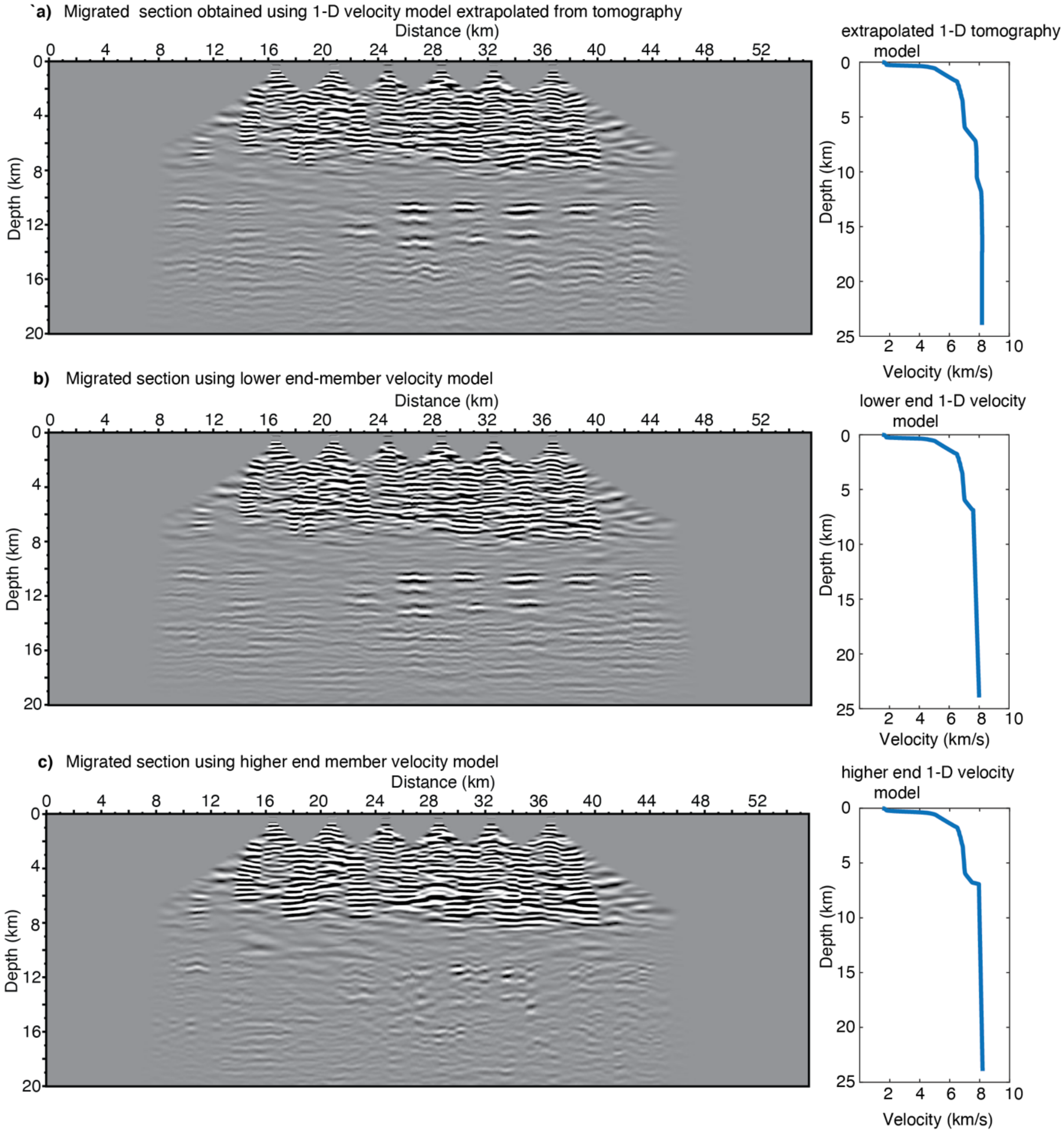

Supplementary Figure 9 | Pre-stack depth migration using different 1-D velocity functions. (a) Pre-stack migrated section is done using the velocity obtained from tomography. **(b)** Pre-stack migration is done using the minimum estimated velocity. **(c)** Pre-stack migration is done using the maximum estimated velocity. The 1-D velocity models are shown in the right of the corresponding pre-stack migrated section. The best image is obtained using the velocity used in this paper.

Thickness =1200 m; velocity increased by 7%

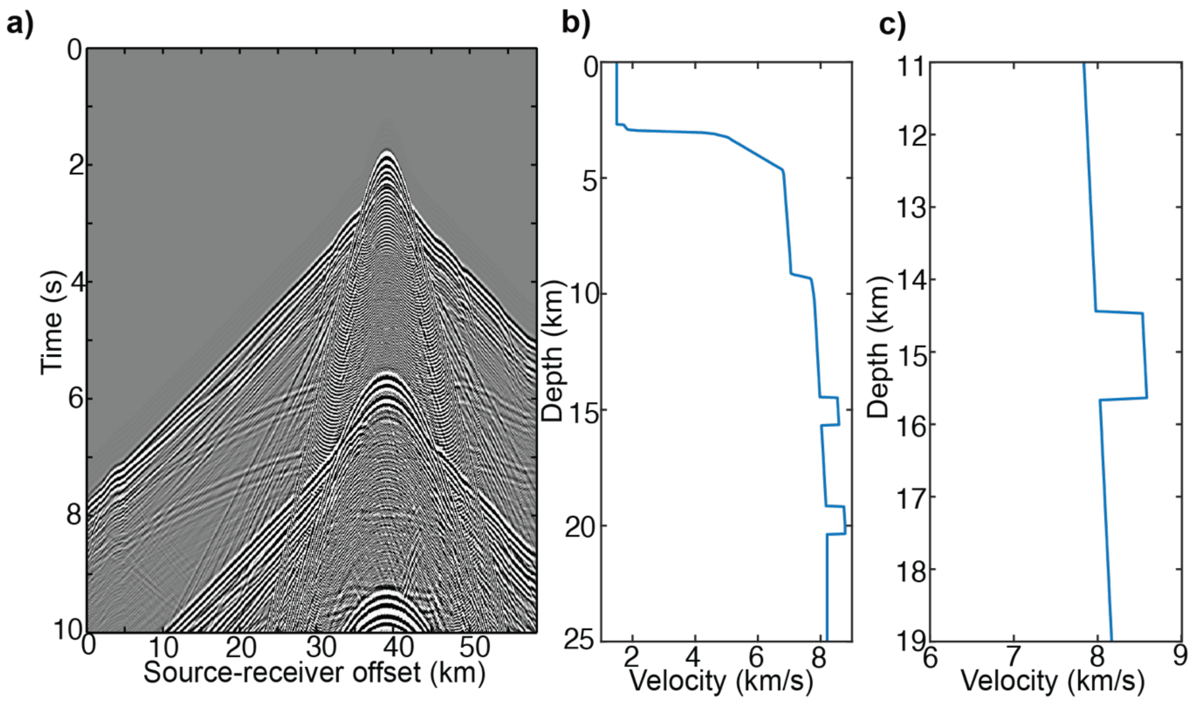

Thickness =300 m; velocity increased by=7%

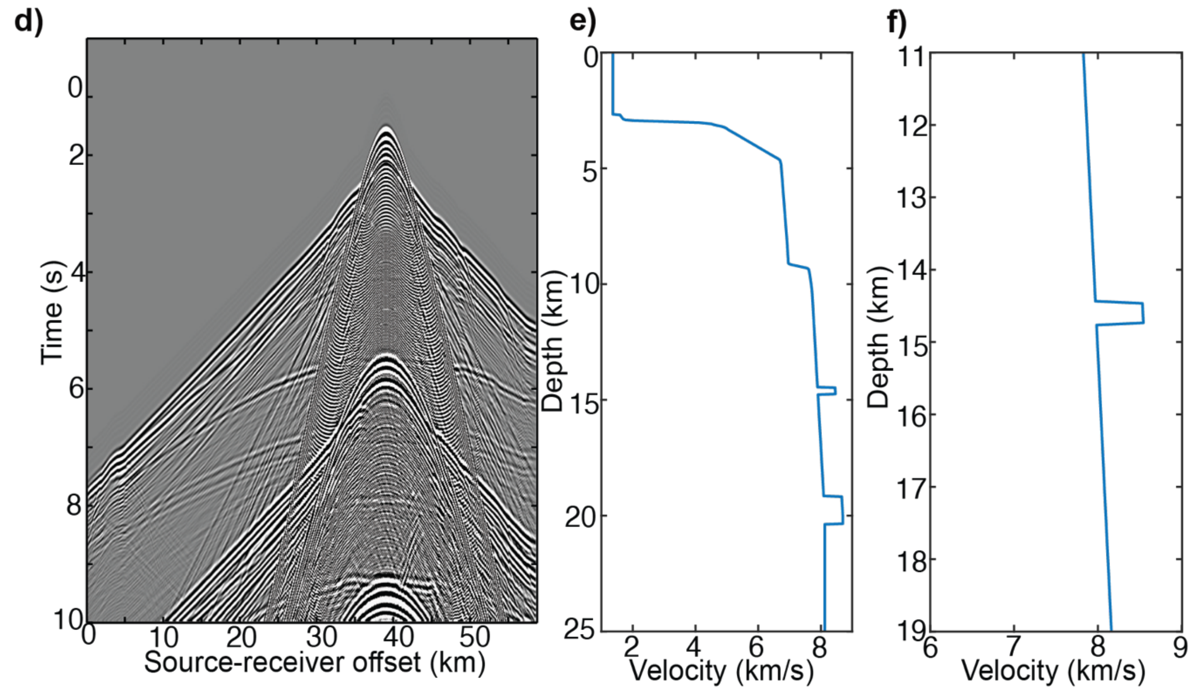

Supplementary Figure 10 | Synthetic modelling to eliminate high-velocity layer. (a) Synthetic seismograms for two 1200 m thick high-velocity layers (**b)** where the layer velocity is +7% higher than the mantle velocity. **(c)** The blow-up of the upper high-velocity layer. **(d)** Synthetic seismograms for a 300 m thick high-velocity upper layer (**e**). **(f)** Blow-up of the upper-velocity layer.

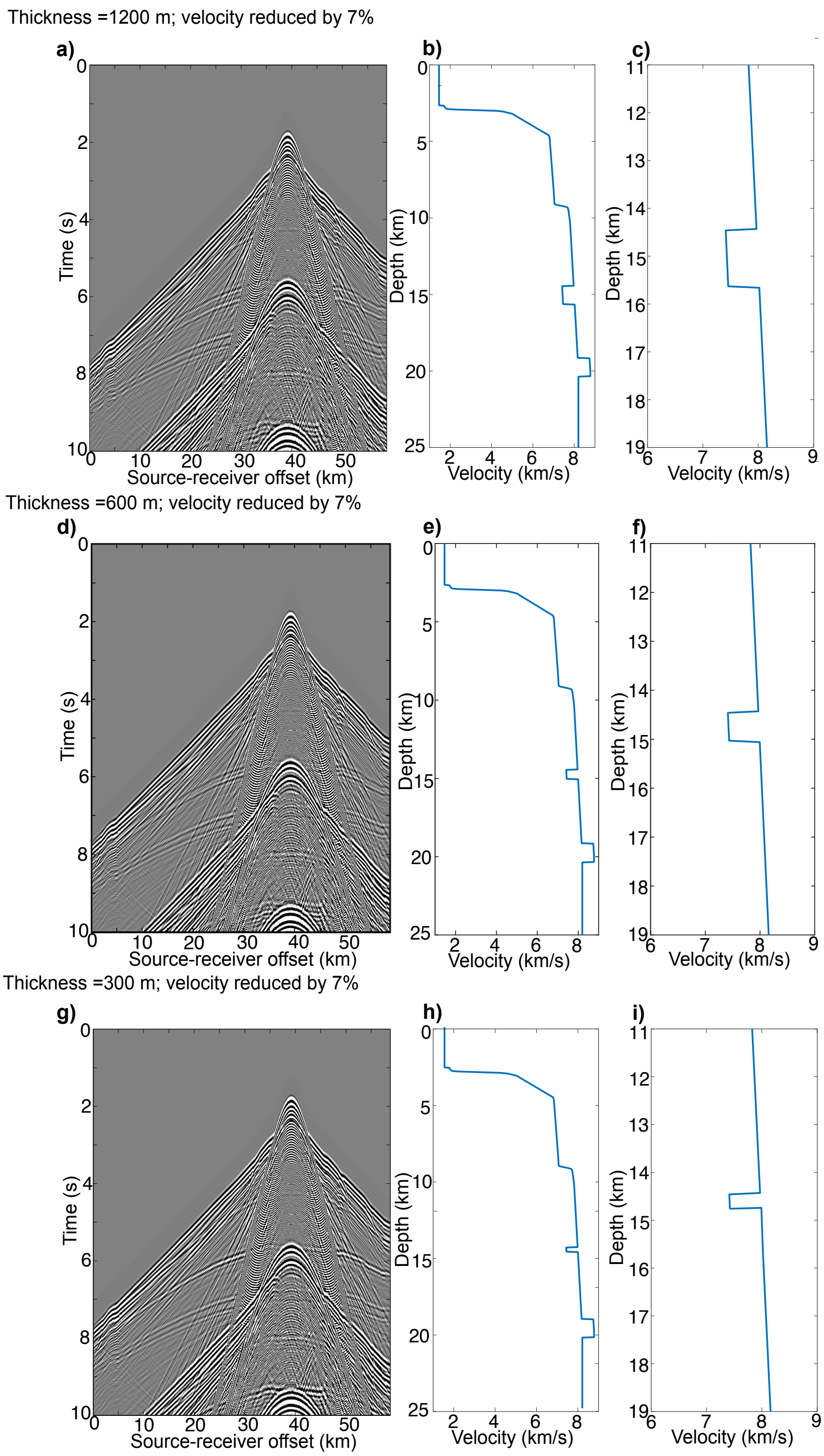

Supplementary Figure 11 | Synthetic modelling to quantify the thickness of the layer with 7% lower velocity. (a) Synthetic seismograms for 1200 m thick lower-velocity layers (b) where the layer velocity is -7% lower than the mantle velocity. **(c)** The blow-up of the upper low-velocity layer. **(d)** Synthetic seismograms for a 600 m thick low-velocity upper layer (**e**). **(f)** Blow-up of the upper-velocity layer. **(g)** Synthetic seismogram for a 300 m thick lower velocity upper layer shown in **(h)** In the panel **(i)** we show a blow-up of the upper lower velocity layer.

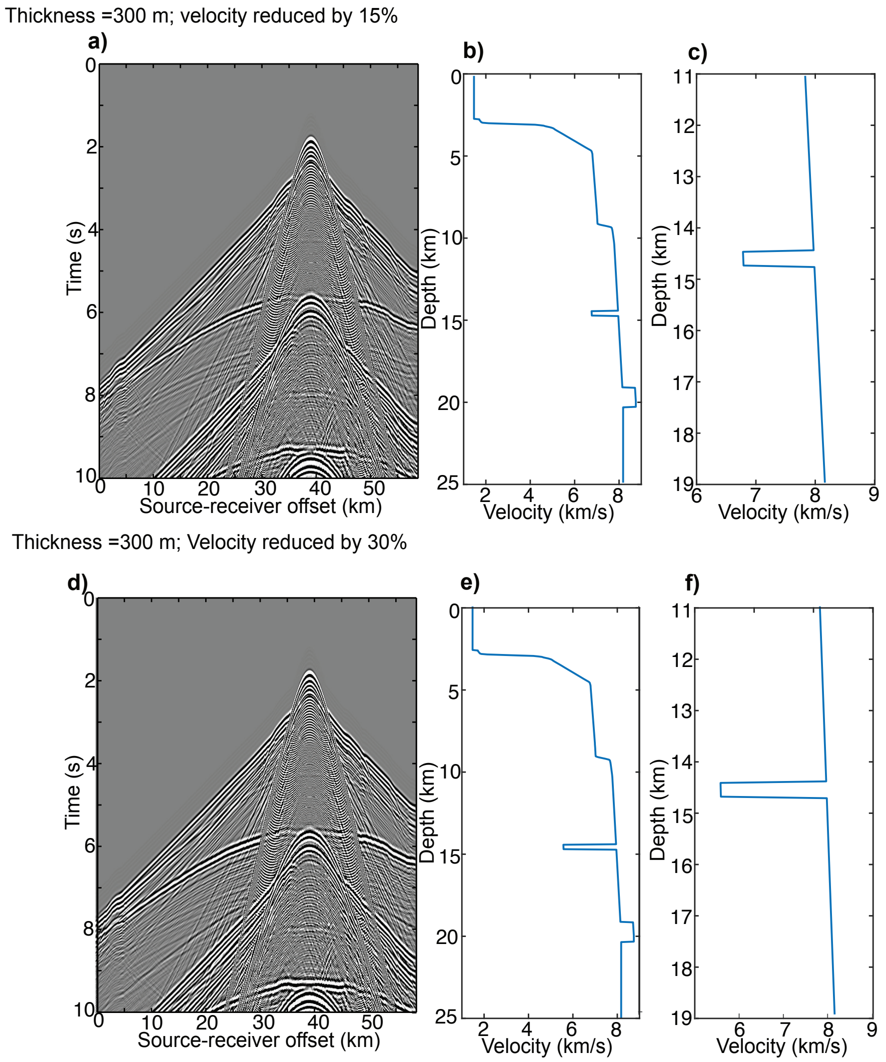

Supplementary Figure 12 | Synthetic modelling to quantify the velocity contrast. (a) Synthetic seismogram for 300 m thick upper layer with a velocity reduced by -15% as shown in **b**. **(c)** Blow-up of the upper-velocity layer. **(d)** Synthetic seismogram for a 300 m thick upper layer with velocity reduced by -30% as shown in **e**. **(f)** Blow-up of the upper lower velocity layer.

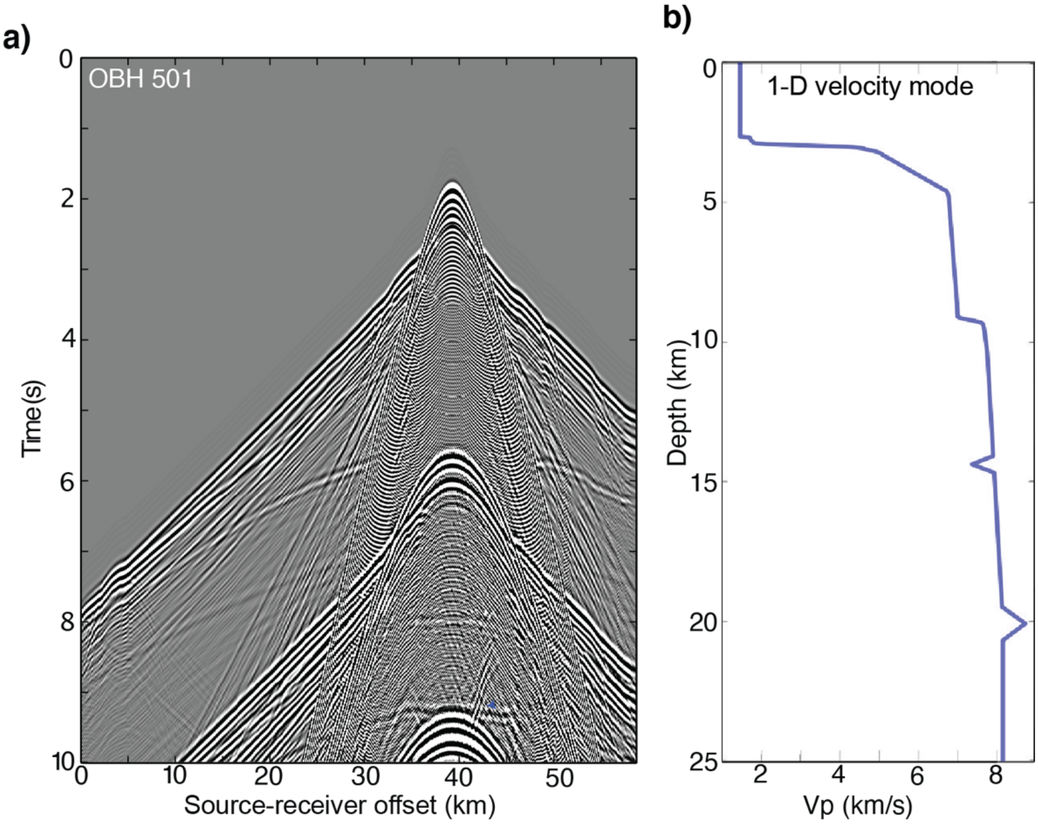

Supplementary Figure 13| Synthetic modelling using gradient layers. (a) Synthetic seismogram obtained by examining the model that includes a shallower, 600-m thick low-velocity layer with velocity linearly increasing and then decreasing and a deeper, 1200-m thick high-velocity layer with velocity increasing and then decreasing. The 1-D velocity model is shown in panel **b**.

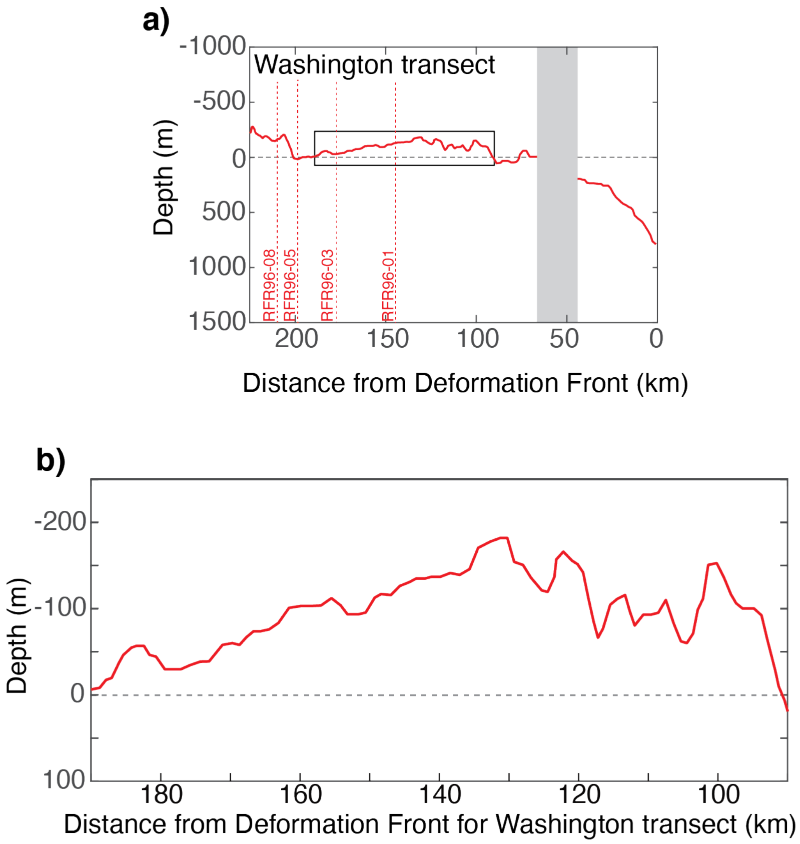

Supplementary Figure 14 | Residual topography. (a) The residual topography is shown for the Washington transect from the JdF Ridge to the deformation front of the Cascadia subduction[2]. The effect of plate cooling is removed, assuming half-space thermal cooling. Predicted subsidence due to sediment loading is taken into account by assuming local isostasy[3] and constant density for sediments (2000 kg/m^3). The black box outlines the region presented in panel **b** that shows the uplift that remained after removing the plate thermal cooling and sediment-loading effects for the Washington transect. In dashed red lines, we show the locations of our OBH profiles. Grey boxes indicate the regions of data gaps.

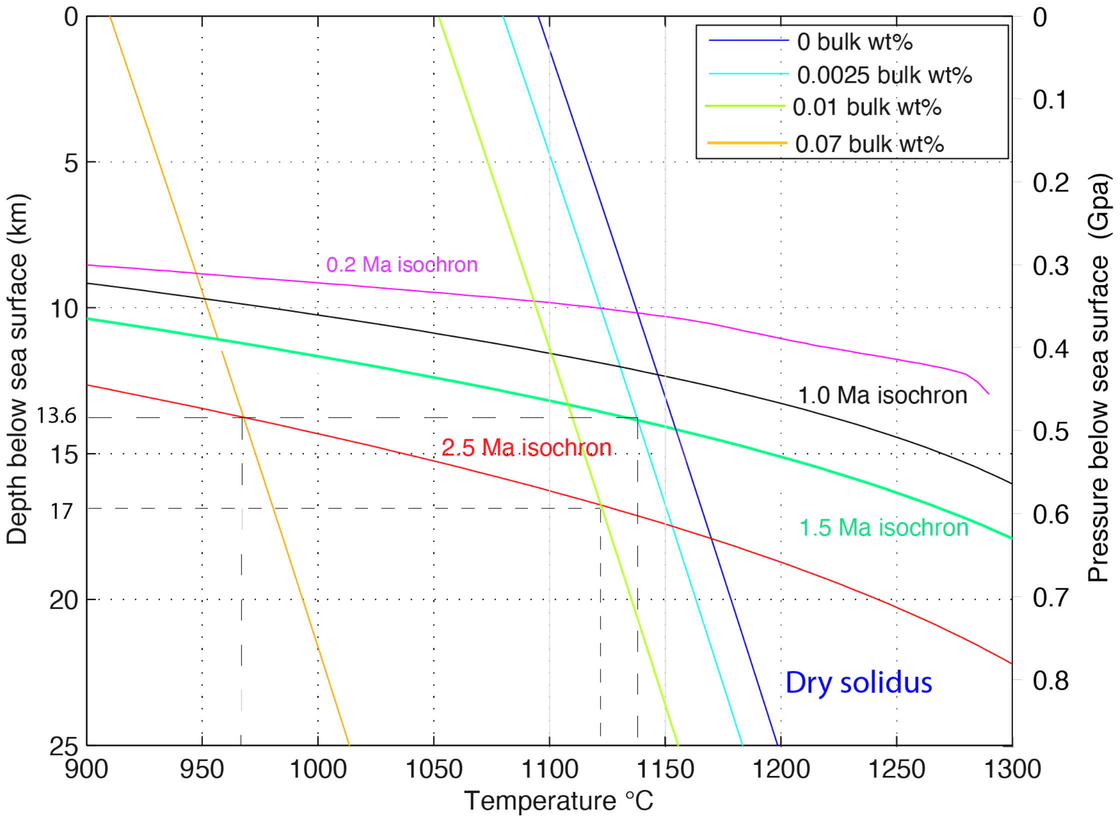

Supplementary Figure 15 | Water content estimation. The range of bulk water content for the solidus system to initiate melting in the mantle. The depth versus temperature relationship at different ages is shown in different coloured curves (1.0 Ma-black, 1.5 Ma-green, 2.5 Ma-red). The intersection points at 13.6 km (11 km below seafloor) for 1.5 and 2.5 Ma are 25 ppm and 700 ppm water content, respectively. Another intersection point at 17 km (14.5 km below seafloor) for 2.5 Ma is 100 part per million (ppm) water content.

References

1. Korenaga, J. et al. Crustal structure of the southeast Greenland margin from joint refraction and reflection seismic tomography. *J. Geophys. Res.* **105**, 21591-21614 (2000).

2. Han, S. et al. Seismic reflection imaging of the Juan de Fuca plate from ridge to trench: New constraints on the distribution of faulting and evolution of the crust prior to subduction. *J. Geophys. Res. Solid Earth* **121**, 1849-1872 (2016).

3. Turcotte, D. L. & Schubert, G. Geodynamics: Applications of Continuum Physics to Geological Problems, John Wiley & Sons, New York, *2nd edition*, P.161 (2002).
